# Blood Transfusion Reactions—A Comprehensive Review of the Literature including a Swiss Perspective

**DOI:** 10.3390/jcm11102859

**Published:** 2022-05-19

**Authors:** Theresa Ackfeld, Thomas Schmutz, Youcef Guechi, Christophe Le Terrier

**Affiliations:** Department of Emergency Medicine, Fribourg Hospital, The University of Fribourg, 1702 Fribourg, Switzerland; theresa.ackfeld@h-fr.ch (T.A.); thomas.schmutz@h-fr.ch (T.S.); youcef.guechi@h-fr.ch (Y.G.)

**Keywords:** erythrocyte transfusion, blood cell transfusion, anemia treatment, adverse transfusion reactions, pulmonary complications

## Abstract

Blood transfusions have been the cornerstone of life support since the introduction of the ABO classification in the 20th century. The physiologic goal is to restore adequate tissue oxygenation when the demand exceeds the offer. Although it can be a life-saving therapy, blood transfusions can lead to serious adverse effects, and it is essential that physicians remain up to date with the current literature and are aware of the pathophysiology, initial management and risks of each type of transfusion reaction. We aim to provide a structured overview of the pathophysiology, clinical presentation, diagnostic approach and management of acute transfusion reactions based on the literature available in 2022. The numbers of blood transfusions, transfusion reactions and the reporting rate of transfusion reactions differ between countries in Europe. The most frequent transfusion reactions in 2020 were alloimmunizations, febrile non-hemolytic transfusion reactions and allergic transfusion reactions. Transfusion-related acute lung injury, transfusion-associated circulatory overload and septic transfusion reactions were less frequent. Furthermore, the COVID-19 pandemic has challenged the healthcare system with decreasing blood donations and blood supplies, as well as rising concerns within the medical community but also in patients about blood safety and transfusion reactions in COVID-19 patients. The best way to prevent transfusion reactions is to avoid unnecessary blood transfusions and maintain a transfusion-restrictive strategy. Any symptom occurring within 24 h of a blood transfusion should be considered a transfusion reaction and referred to the hemovigilance reporting system. The initial management of blood transfusion reactions requires early identification, immediate interruption of the transfusion, early consultation of the hematologic and ICU departments and fluid resuscitation.

## 1. Introduction

Although blood transfusions can be a life-saving therapy, every transfusion carries a substantial risk of adverse reactions. In Switzerland (CH), 275,343 blood products were transfused in 2020, and of these 212,947 were red blood cells (RBC), 35,715 were platelet concentrates (PC) and 26,681 were fresh frozen plasma (FFP). For all blood products, a total of 2032 transfusion reactions were reported, of which 1910 were imputed as at least “possible”. In Switzerland, imputability is considered as follows: 0: not assessable, 1: unlikely, 2: possible, 3: probable, and 4: certain. Of these possible transfusion reactions, 78% were classified as severe or above [1]. Additional data concerning the classifications of blood transfusion reaction imputability and severity in Switzerland, France (F), United Kingdom (UK) and Germany (D) are displayed in the Supplementary Material (Supplementary S1, Tables S1–S5). Despite the postponement of elective surgeries due to the COVID-19 pandemic, many blood transfusions still had to be performed in 2020. However, in Switzerland, the previously observed trend continued, with the number of transfusions decreasing by almost 4% compared to the previous year. Despite this trend, the number of hemovigilance reports increased by 18% compared to 2019, highlighting the rising concern about transfusion reactions as well as the sensibilization of medical staff to the importance of hemovigilance. This trend is consistent with the years before COVID-19. For example, in 2017 in Switzerland, 293,069 blood products (226,276 RBC) were transfused with 1223 (imputability 1–4) reported transfusion reactions, whereas in 2018, 1590 (imputability 1–4) transfusion reactions were reported for 290,599 transfused blood products (221,100 pRBC) [1,2,3].

Blood transfusion, even though it is a common procedure in medical settings, remains an invasive medical act that should not be underestimated. It is imperative that the physician and the administrator of blood products are aware of the appropriate administration, the hazards of complications and the risks of the procedure. The aim of this article is to provide an overview of transfusion complications, to discuss the management of each transfusion reaction according to the current literature and to compare the epidemiology of Switzerland, France, Germany and the United Kingdom in terms of executed transfusions, reporting rates, transfusion reactions and mortality rate per reported incidence.

## 2. Review Design and Methods

An electronic search in the “PubMed” database from 1943 to 2022 was performed. The following terms were used in the search strategies: “Transfusion reaction”; “Acute hemolytic transfusion reaction”; “Febrile non-hemolytic transfusion reaction”; “Anaphylactic transfusion reaction”; “Minor allergic transfusion reaction”; ”Transfusion-associated circulatory overload”; ”Transfusion-related acute lung injury”; “Massive transfusion-associated complications”; “Septic transfusion reaction”; “Bacterial contamination blood transfusion” and “COVID RBC transfusion” in the title and abstract. All articles in the English, German and French languages were scanned. Furthermore, official hemovigilance reports from Switzerland, France, Germany and the UK were evaluated. Exclusion criteria were articles that were written in any language other than English, French or German. Articles were not included if in the original article was not available in “PubMed”. We focus here on acute transfusion reactions; delayed transfusion reactions and adverse events in the donor were not considered in this review. Transfusion-related viral infections, with exception of COVID-19, were also excluded.

## 3. Transfusion Reactions

### 3.1. Epidemiology

In Switzerland, 275,343 blood products (packed red blood cells, platelet concentrates and fresh frozen plasma) were transfused in 2020, and 2032 (0.74%) reports of adverse transfusion reactions were evaluated. Of these, 1910 (0.69%) reactions were classified as possible transfusion reactions (imputability 2–4) and 1486 (0.54%) were classified as severe or above (grade 2–4, Supplementary S2), with 3 deaths (0.001% mortality per transfused blood product) [1]. In France, in comparison, 2,806,774 blood products were transfused in 2020. The Agence nationale de sécurité du médicament et des produits de santé (ANSM) received 9060 (0.32%) reports of adverse transfusion reactions in recipients in total, of which 7062 (0.25%) were classified as possible transfusion reactions (imputability 1–3) and 610 were classified as severe or more (grade 2–4), with 5 deaths (0.0002% mortality per transfused blood product) [4]. The German Paul Ehrlich Institute (PEI) reported the transfusion of 4,400,164 blood products in 2020, with 921 (0.02%) suspected cases of serious adverse transfusion reactions. In this case, no detailed information on the imputability was given in the report, but for 621 (0.014%) cases a causal relationship with the administration of blood components was confirmed by the PEI, with 7 deaths (0.0002% mortality per transfused blood product) [5]. In the UK, 2,074,517 blood products were issued in 2020, with a total of 4063 (0.2%) submitted reports, of which 2881 (0.14%) were included in the 2020 report. Unfortunately, no information about imputability was available in these 2881 reports. There were 39 deaths (0.002% mortality per transfused blood product) related to transfusion in the UK in 2020 (imputability 1–3) [6,7].

Mortality, defined as deaths per total number of transfusions, differs between the different countries, but so does the reporting rate of transfusion reactions. Table 1 provides an overview of the total numbers of blood transfusions and transfusion reactions, and their imputability and severity, as well as the mortality per transfused blood product, the reporting rate of adverse transfusion reactions and the death rate per reported event. Germany especially seems to underestimate the rate of minor adverse events, since hemovigilance reporting is only required for serious reactions and the PEI only sporadically receives information on non-serious events, so that these were not included in their evaluation [5].

The numbers presented here for different countries should, however, be evaluated carefully. Not only do the reporting systems and the designations of imputability differ from one country to another but also the definition of adverse events. In Germany, for example, a febrile non-hemolytic transfusion reaction (FNHTR) is defined as a fever ≥39 °C with an increase of ≥2 °C compared with the value before transfusion, versus ≥38 °C with an increase of ≥1 °C following the definition of the National Healthcare Safety Network (NHSN) [5,8]. Other authors also noted great variability in transfusion-associated adverse event rates in different countries, mostly due to passive surveillance, different definitions of each transfusion reaction and the use of different blood products, underlining the difficulty of interpretation of these hemovigilance data [9]. The data presented here are displayed in Table 1. Supplemental data from Switzerland, France and Germany are provided in Supplementary S2, Tables S1–S4.

### 3.2. Definition of Blood Transfusion Reactions and Initial Management

Transfusion reactions are defined as adverse events following a blood transfusion, with the severity ranging from minor to life-threatening. In a clinical setting, any new symptom or change in vital signs occurring within 24 h of a blood transfusion should be considered a transfusion reaction until proven otherwise [10]. The diagnosis is often difficult to establish, due to a wide range of symptoms that are mostly overlapping. Each adverse event following a blood transfusion should be considered severe until further work-up is performed.

**Figure 1:**
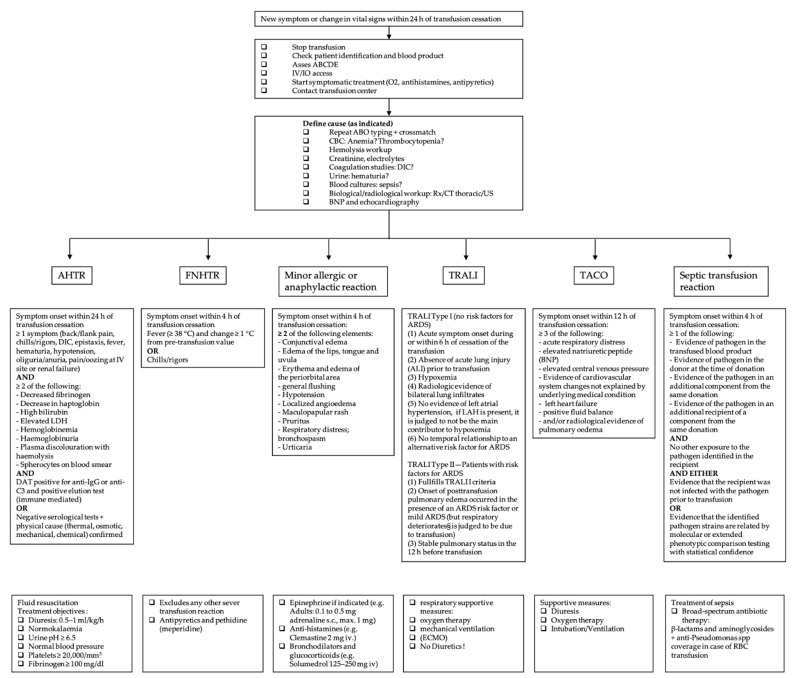
Overview of the most common acute transfusion reactions with treatment propositions. CBC = complete blood count; DIC = disseminated intravascular coagulation; BNP = brain natriuretic peptide; AHTR = acute hemolytic transfusion reaction; FNHTR = febrile non-hemolytic transfusion reaction; TRALI = transfusion-related acute lung injury; TACO = transfusion-associated circulatory overload; LDH = lactate dehydrogenase; ARDS = acute respiratory distress syndrome; LAH = left atrial hypertension; RBC = red blood cell.

The symptomatic patient should be evaluated immediately. Initial management for all types of transfusion reactions includes stopping the transfusion, keeping the intravenous line open, providing supportive and symptomatic therapy and checking the patient identification and the blood product labeling [11,12]. Figure 1 gives an overview of the definition and specific management of each transfusion reaction according to the current literature.

**Table 1:** Epidemiology of transfusion reactions (TR) in Switzerland, France, Germany and the United Kingdom.

Region	Transfused Blood Products in 2020	Reported TR	Imputability	Severity Grade 2–3	Deaths	Mortality (Death/Transfused Blood Product)	Reporting Rate	Death/Reported TR
CH	275,343	2032	1910 ^1^	1486	3	0.001%	0.74%	0.14%
F	2,806,774	9060	7062 ^2^	610	5	0.0002%	0.32%	0.06%
D	4,400,164	921	621 ^3^	n/a	7	0.0002%	0.02%	0.76%
UK	2,074,517	4063	2881 ^4^	n/a	39	0.002%	0.2%	0.95%

^1^ Imputability 2–4 according to classification of Switzerland; ^2^ Imputability 1–3 according to classification of France; ^3^ No detailed information on the imputability was given but a causal relationship with the administration of blood components was confirmed by the institute; ^4^ Reports included in the final report, but no information on imputability was given. All classifications are displayed in the Supplementary Material (Supplementary S1, Tables S1–S5).

### 3.3. Acute Hemolytic Transfusion Reaction

In 2020, in Switzerland, hemolytic transfusion reactions accounted for 1 per 10,000 transfusions, with 22 cases classified as severe or above [1].

Immune-mediated acute hemolytic transfusion reactions (AHTR) are most often related to ABO incompatibility but can also be caused by non-ABO antigens (e.g., irregular antibodies, anti-K, 1 anti-Fya, 1, anti-Jkb, mixed antibodies including anti-Jka, anti-Jkb and anti-Jk3 and anti-E and anti-K). The extent of hemolysis, and therefore the severity of the reaction, depends on multiple factors such as the involved immunoglobulin class, subclass and antibodies. Pathophysiological AHTR involves intravascular or extravascular hemolysis, with or without complement activation. As the expression of ABO antigens on RBC is higher than other antigens, more antigen–antibody complexes are formed, and therefore more sites for complement activation are present. This may explain the severity of ABO incompatibility. Other reasons are the lower titers of irregular antibodies and the dilution in the recipient’s plasma. The volume of the ABO-incompatible blood product may also play a role. A higher mortality is associated with transfused volumes over 200 mL, yet fatal blood transfusions have also been reported with small volumes (25 mL), especially in pediatric patients. Laboratory parameters, however, do not predict the severity [13,14,15].

Despite no clear data being reported, immunological incompatibility seems to be the most frequent cause of hemolytic transfusion reactions, generally caused by misidentification of the patient or the blood sample at the time of collection or transfusion [14]. Therefore, careful pre-transfusion testing is indispensable, in order to match RBC donors and recipients and to prepare immunologically compatible blood products.

Of special importance in the emergency department is the emergency transfusion of non-compatible blood products, which is practiced in trauma centers worldwide. Nevertheless, there is a small but potentially serious risk of acute hemolytic transfusion reactions (<1/1000) that the clinician should be aware of [16].

On the other hand, non-immune-mediated reactions are the result of red blood cell destruction due to mechanical, thermal, chemical or osmotic damage [14].

Symptoms of AHTR usually occur within 24 h of the transfusion [8]. Although the classic triad involves fever, kidney pain and hemoglobinuria, the symptoms can be very varied and non-specific [14]. Patients may present with pruritus, jaundice, hypotension, tachycardia, tachypnoea, pain (at the side of the veinous access or in the renal compartment), nausea, disseminated intravascular coagulation, acute renal failure, shock and even death [8]. Biologically, two of the following elements must be found: a decrease in fibrinogen or haptoglobin, an increase in bilirubin or LDH, hemoglobinuria and hemoglobinemia leading to plasma discoloration or spherocytes on the blood smear. In addition to this work-up, an immune analysis with repeat crossmatching, grouping and an elution test should be performed [8]. Since fever and chills may be the only early signs, it is important to stop the transfusion immediately and begin the diagnostic investigation.

AHTR is a medical emergency. There is no specific treatment, and management consists mainly of fluid resuscitation with a target diuresis of 0.5–1 mL/kg/h. Treatment objectives are normokalaemia, urine pH ≥ 6.5, normal blood pressure, platelets ≥ 20,000/mm^3^ and fibrinogen ≥ 100 mg/dL [14]. Some authors propose steroids, plasma exchange and continuous hemodiafiltration, and others propose immunoglobulins or complement inhibitors. The pathophysiological mechanism behind this is the elimination of cytokine release in the early phase of AHTR via steroids or plasma exchange (after incompatible blood transfusion plasma exchange therapy removes anti-A or anti-B antibodies, which inhibit the antigen–antibody reaction, and free hemoglobin is also removed, which inhibits disseminated intravascular coagulation and acute kidney injury) or cytokine action, for example, via JAK-STAT inhibitor Ruxolotinib or via Eculizumab, a monoclonal antibody against complement 5 that inhibits the formation of membrane attack complex [17,18,19]. One case study could show that Eculizumab successfully inhibited hemolysis for several weeks after the transfusion of major ABO-incompatible RBCs [19]. The possibly life-saving use of Ruxolotinib, Eculizumab, continuous hemofiltration and plasma exchange in patients with a severe course, however, has only been shown in case studies. Even in 2022, there is no scientific evidence available to support these attitudes, and additional therapies should be decided on a case-by-case basis. In any case, the transfusion center and the intensive care unit should always be informed as soon as possible.

### 3.4. Febrile Non-Hemolytic Transfusion Reaction (FNHTR)

FNHTR, with an incidence of 1.44 per 1000 transfusions in 2020 in Switzerland, is a frequent complication [1]. It is characterized by a fever (+>1 °C or >38°) without hemolysis, which may be accompanied by chills, tachypnoea, anxiety, headache, transient hypertension and discomfort within 4 h of transfusion [8,12,20]. Two etiologies are described. In immune-mediated FNHTR, the symptoms are attributable to the release of endogenous pyrogens from white blood cells (WBCs) (either from the patient or the recipient), following a reaction between the recipient’s antibodies and the donor’s antigens [21]. Non-immune-mediated FNHTR is described by the release and accumulation of pro-inflammatory cytokines by WBCs in the blood product during storage. Critical factors are thought to be WBC content and age [20].

The diagnosis is one of exclusion. It is therefore necessary to rule out any other cause requiring urgent management such as acute hemolytic transfusion reaction.

When the clinical and biological work-up (hemolysis work-up, administrative verification, ABO confirmation and direct antiglobulin test) excludes any other severe transfusion reaction, symptomatic treatment with antipyretics can be started. For severe rigor, a treatment with pethidine may be tried [20,22]. When used, 25 mg of Demerol in a slow intravenous push is recommended as a first dose, with an additional 25 mg 10 to 15 min later if rigor persists [23,24]. The treatment approach remains a symptomatic one, and little information on treatment is found in the literature. Prevention of FNHTR is nevertheless an important subject. The efficacy of preventative premedication remains controversial, and routine premedication with acetaminophen and antihistamines in clinical studies did not prevent nonhemolytic transfusion reactions. Leukodepletion and plasma reduction of platelet and blood components, however, may play an important role, even though this seems to reduce the occurrence of FNHTR by only half or less [25,26,27]. Clinical studies to identify the most effective prevention approach have not yet been reported, and further research is mandatory.

### 3.5. Anaphylactic Transfusion Reaction (ATR) and Minor Allergic Transfusion Reaction

ATRs and minor transfusion reactions are type 1 hypersensitivity reactions, accounting for 9% of all possible transfusion reactions in Switzerland in 2020 [1]. The severity of these reactions varies from simple skin and mucous membrane damage to upper and lower airway and cardiovascular system involvement. The diagnosis is clinical. Symptoms appear within 4 h of the transfusion and are related to the release of histamine from mast cells and basophils [28]. They do not differ from those of other allergic reactions, and the therapeutic management is superimposable on other anaphylactic reactions [8]. Tryptase blood level can help to confirm the diagnosis but does not rule it out if it is negative (half-life of 2 h). A basophil activation test (BAT) performed with residual transfused blood and the patient’s own blood often confirms the allergic reaction [28]. A simple mucocutaneous reaction does not contraindicate future transfusions.

### 3.6. Lung Transfusion Complications

Transfusion-related acute lung injury (TRALI) and transfusion-associated circulatory overload (TACO) are serious, life-threatening pulmonary transfusion reactions. Despite the parallels between TACO and TRALI, it is important to distinguish these two diagnoses as their treatment and prevention differs considerably.

### 3.7. Transfusion-Related Acute Lung Injury (TRALI)

TRALI was cited in 0.15% of hemovigilance reports in 2020 in Switzerland. This incidence is, however, probably underestimated [1]. Although the reported incidence of TRALI is low, mortality is high; the Food and Drug Administration (FDA) reported a transfusion-associated fatality rate of 27% due to TRALI in the fiscal year 2019, highlighting the importance of recognizing this complication [29].

One reason for this underestimation is a lack of understanding among clinicians, especially due to the difficulty of distinguishing TRALI from other entities. The main differential diagnosis is acute respiratory distress syndrome (ARDS). Several classifications have been developed in order to distinguish TRALI from ARDS and to provide accurate data on adverse transfusion reactions [30,31].

TRALI is an acute non-cardiogenic pulmonary oedema associated with hypoxemia. It was initially classified by the 2004 definition of TRALI and possible TRALI (pTRALI) at the Canadian Consensus Conference CCC [30]. In this definition, patients who present with symptoms of TRALI but who also have ARDS risk factors are classified as pTRALI, to underline the fact that ARDS cannot be excluded. In 2019, these definitions, as well as those for TRALI including criteria for diagnosis, clinical findings, timing of onset and relationship to ARDS risk factors were reconsidered, and a more clinical approach was advocated [31]. This new definition dropped the term pTRALI and defines TRALI type I as new, acute respiratory distress within 6 h of blood transfusion in the absence of temporally associated risk factors for ARDS. The definition is based on five mandatory criteria: (1) absence of acute lung injury prior to transfusion; (2) occurrence of acute lung injury during or within 6 h of cessation of transfusion; (3) hypoxemia; (4) radiographic evidence of bilateral lung infiltrates; and (5) no evidence of left atrial hypertension (LAH) or if LAH is present, it is judged to be not the main contributor to the hypoxemia [8,31]. TRALI type II is defined by three criteria: (1) it must fulfill the same clinical criteria as TRALI type I; (2) the onset of post-transfusion pulmonary edema occurred in the presence of an ARDS risk factor or mild ARDS; and (3) there was a stable pulmonary status in the 12 h before transfusion [31].

TRALI remains a clinical diagnosis, and the clinician’s judgement plays an important role. However, patients with risk factors who develop ARDS within 6 h of transfusion and who already had pulmonary deterioration 12 h before transfusion should be considered as displaying ARDS and not TRALI [32].

Pathophysiologically, TRALI is most often regarded as an immune-mediated reaction based on the “two-hit” hypothesis that has been repeatedly replicated in animal models [33]. The “first hit” corresponds to the development of a systemic pro-inflammatory state such as sepsis, surgery or massive transfusion of blood products [32]. The “second hit” is regarded as neutrophil activation with release of reactive oxygen species that damage the pulmonary vasculature. As a result, pulmonary capillaries become permeable and extravasation of fluid into the pulmonary interstitium causes non-cardiogenic pulmonary oedema. A small percentage of cases occur without immune mediation and are the result of biologically active lipids in donor plasma, most often associated with stored platelets and RBCs [32].

The cornerstone of TRALI management is respiratory support measures (oxygen therapy or even mechanical ventilation with a protective lung ventilation strategy), with some patients requiring extra-corporeal membrane oxygenation (ECMO) [34]. In contrast to TACO, diuretics may be harmful by causing hypotension and should be avoided [34]. Although effective in ARDS, no clear evidence of benefits from corticosteroid therapy in TRALI have been found [34,35]. With regard to treatments such as corticosteroids, albumin and statins, the literature is currently not sufficient to support the attitude [36,37,38]. Although IL-10 therapy, anti-complement agents and anti-platelet agents have been investigated in animal models, the most promising therapeutic strategies seem to be IL-10 therapy, CRP downregulation, targeting reactive oxygen species (ROS) or blocking IL-8 receptors [39,40,41,42]. Moreover, in high-risk patients, new leukoreduction technologies and product washing may improve outcomes [34].

### 3.8. Transfusion-Associated Circulatory Overload (TACO)

In Switzerland, in 2020, TACO was the leading cause of morbidity and mortality related to blood transfusion, with 88 reported cases, of which 27 were classified as life-threatening or fatal [1]. Data from other countries support this trend. The UK reported TACO (together with transfusion delays) as the most common cause of transfusion-related deaths in 2020, accounting for 30/39 deaths (76.9%) [6]. Accordingly, in 2019, the FDA classified TACO (together with TRALI) as the most common cause of transfusion-related deaths, at 27% [29]. These numbers highlight the importance of the management and prevention of TACO.

According to the latest version of the National Healthcare Safety Network (NHSN), TACO is defined as the new onset or exacerbation of respiratory symptoms within 12 h of transfusion. This changed from the 2016 definition, which specified symptom onset within 6 h. Three or more of the following must be present: acute respiratory distress, elevated natriuretic peptide (BNP), elevated central venous pressure, left heart failure, positive fluid balance and/or radiological evidence of pulmonary oedema [8]. This definition is also supported by the International Society of Blood Transfusion [43]. TACO is related to circulatory overload, but several studies suggest additional pathophysiological factors [32,41]. The pathophysiological mechanism of TACO is still incompletely understood, and as in TRALI, a “two-hit” theory is proposed [41]. The 2017 study by Parmar et al. highlights a new fever in one third of patients, suggesting other components such as pro-inflammatory reactions that deserve further investigation [44]. A further hint on pathogenesis may be the finding of a 2010 study showing a 50% decrease in the occurrence of TACO with the introduction of universal leukoreduced products [45]. Further studies are needed to shine light on the role of inflammation in TACO. Prevention of TACO and identification of high-risk patients is essential. According to a 2013 retrospective study, the risk factors for developing TACO are a history of heart failure (41%), renal failure (44%) and age over 70 years (65%), and special attention should be played to these patient groups [32]. Swiss data from 2020 also suggest that TACO was mostly observed in the high-risk group (>70 years), with 54 cases of TACO out of a total of 88 cases. This may be due to the unadjusted transfusion rate in the presence of risk factors [1]. Identifying patients at risk and the use of slower transfusion rates in selected patients, as well as prophylactic volume reduction with diuretics, may be beneficial [46,47]. At present, there is no causal treatment, and the management of TACO is similar to that of acute heart failure, consisting of diuresis, oxygen and ventilation or intubation if needed [41].

### 3.9. Massive Transfusion-Associated Complications

No universal definition for massive transfusion (MT) is found in the current literature, but the persistent transfusion requirement of >4 packed red cells (approximately 1000 mL) within 1 h or the transfusion of 10 units of packed red blood cells within a 24 h period is a commonly accepted definition in clinical settings [48].

Complication prevention starts with the correct administration of blood products when MT is indicated. Several scores have been developed in order to predict clinical situations that warrant MT, such as the German Trauma-Associated Severe Hemorrhage (TASH) score, with a correct classification rate of over 90%, the Prince of Wales Hospital/Rainer score (PWH score) with a correct classification rate of 97%, the American Vandromme score with a positive predictive value of 75% and many more [49,50,51]. The TASH score, however, is the most well-validated score. The choice of score needs to be individualized, considering the available skill set as well as hospital resources [52].

**Figure 2:**
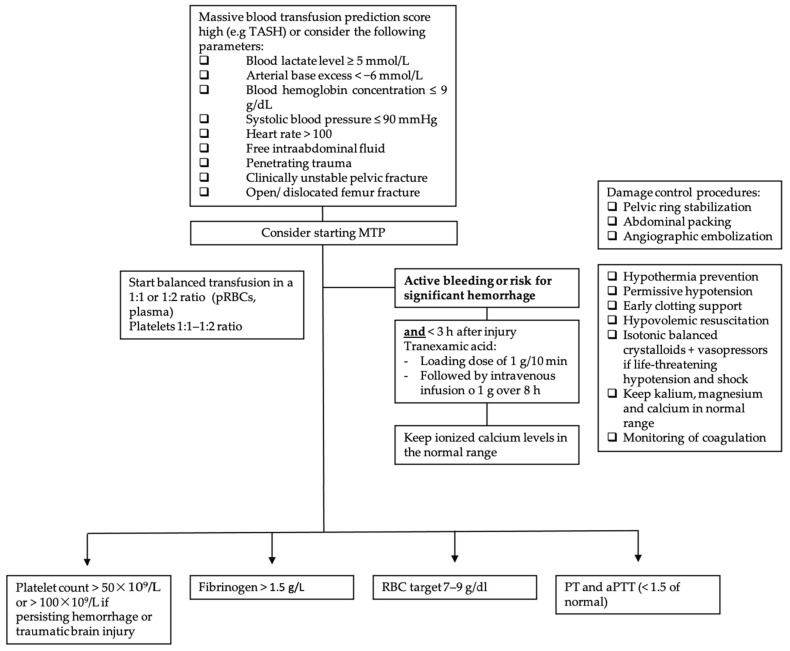
Massive transfusion protocol algorithm. TASH score = Trauma Associated Severe Hemorrhage; RBC = red blood cell; pRBC = packed red blood cell; PCC = prothrombin complex concentrate (PCC); PT = prothrombin time; aPTT = activated partial thromboplastin time; MTP = massive transfusion protocol.

Furthermore, massive transfusion protocols (MTPs), which are used in most trauma centers, help to standardize the resuscitation approach in traumatic hemorrhagic shock. Figure 2 displays the following information in a flow chart. MTPs provide guidance to the blood bank on the use of blood products and are associated with reduced blood utilization and improved outcomes [53,54,55]. Indications to start the MTP are as follows: blood lactate level ≥ 5 mmol/L; arterial base excess (BE) < −6 mmol/L; blood hemoglobin (Hb) concentration ≤ 9 g/dL; systolic blood pressure (SBP) ≤ 90 mmHg [56]. Although MT improves patient outcome, it is associated with various complications. In addition to the general adverse transfusion reactions described in this review, patients receiving MT are especially prone to developing coagulation abnormalities, hypothermia and acidosis. Hypoperfusion and lactate release by RBC during storage, as well as by sodium citrate (an anticoagulant used in stored blood products), further enhance these complications. Hyperkalemia, on the one hand, can occur due to high potassium levels in stored blood products, and cases of hyperkalemic cardiac arrest have been described, especially associated with critically ill patients and fast transfusion rates exceeding 100–150 mL/min [49,57]. Hypokalemia, on the other hand, can develop due to metabolic alkalosis following citrate administration, as well as the use of potassium-poor solutions including crystalloid solutions, platelets and fresh frozen plasma (FFP) [49]. Calcium and magnesium can bind to citrate, and this is used for anticoagulation of the blood products, and ionized calcium levels (total serum calcium concentrations should not be used because of hemodilution, which occurs during resuscitation) and magnesium levels should be monitored and kept in the normal range [49]. In addition, it is important to be aware of increasing bacterial infections in MT patients due to transfusion-related immunomodulation [58]. 

Post-traumatic hemorrhage, however, is the major cause of death in patients who sustained severe trauma and is generally attributable to two mechanisms: bleeding caused by the direct injury of blood vessels and bleeding due to trauma-induced coagulopathy (TIC) [59]. Approximately one third of patients who receive MT present TIC. TIC is caused by three variables: acute traumatic coagulopathy (ATC), coagulopathy induced by resuscitation maneuvers and detrimental factors such as acidosis, hypothermia, shock, male sex, comorbidities, genetic background, inflammation and premedication, e.g., oral anticoagulants [59]. The pathophysiology of ATC is multifactorial due to protein C activation, endothelial glycocalyx disruption, consumption of fibrinogen and platelet dysfunction, but improper medical management can worsen the outcome [60]. TIC was initially thought to be caused solely by the dilution of clotting factors due to massive transfusion and fluid resuscitation. This was thought to enhance the development of acidosis and hypothermia, also known as the “lethal triad” [12,59]. Newer research, however, has shown that TIC appears early in trauma, before medical intervention, acidemia or hypothermia occurs. ATC and coagulopathy induced by resuscitation can coexist, but pathogenies must be distinguished [59].

When MT protocols are indicated, the RBC target is 7–9 g/dL. Supportive measures such as hypothermia prevention, permissive hypotension, early clotting support, hypovolemic resuscitation and isotonic balanced crystalloids with vasopressors in cases of life-threatening hypotension and shock are the cornerstones of therapy [59]. In order to uncover TIC, early monitoring of coagulation is imperative. When an increase in aPTT, PT and INR is observed (PTT or aPTT > 1.5× normal value), FFP or coagulation factor concentrates (PPCs) are indicated, although PPCs have been proven to be better than FFP for rapidly reversing vitamin K antagonists [59]. Fibrinogen supplementation should be started when under 1.5 g/L (Clauss method), with a suggested initial dose of 3–4 g or 50 mg/kg of concentrated fibrinogen. Platelet concentrates are indicated with a target value of >50 × 10^9^/L or >100 × 10^9^/L in cases of persisting hemorrhage or traumatic brain injury [59].

### 3.10. Septic Transfusion Reaction

Septic transfusion reactions, mostly represented by transfusion-transmitted bacterial infections (TTBI), accounted for 0.2% of all transfusion reactions classified as at least possible, and 2.1% of all reported transfusion reactions in Switzerland in 2020 [1]. In France and in the UK, no TTBIs were reported in 2020, compared to 2 events in 3 million and 1 event in 2 million of all transfusions in 2019, respectively [4,6]. In Germany, 2,4 events per million (of all transfusions) were declared in 2020 [5]. A wide spectrum of microorganisms can be associated with TTBI, including Gram-positive bacteria (*Bacillus cereus*, *Enterococcus* spp., *Coagulase-negative staphylococcus* or *Cutibacterium acnes*) and Gram-negative bacteria (*Klebsiella* spp., *Serratia* spp., *Acinetobacter* spp., *Pseudomonas* spp., *Enterobacter* spp., or *Yersinia enterolitica*). Most of those pathogens are skin, enteric or environmental microorganisms [61,62,63]. Platelets stored at room temperature are more susceptible to contamination than red blood cells, and the incidence of contamination is estimated to be between 1:1000 and 1:2500 [64]. This great variability in septic transfusion reaction rates is attributable to different surveillance measures, with passive surveillance missing most contamination events [65]. Because of their storage at −25 °C, plasmas are not implicated in transfusion-transmitted bacterial infections [66].

Septic transfusion reactions occur within 4 h of transfusion with fever, hypotension, chills and other signs of bacterial infection (qSOFA criteria) [12].

When post-transfusion bacterial infection is suspected, bacterial samples should be taken from the patient and from every transfused blood product (culture and Gram stain). Definitive diagnosis requires isolation of the same microorganism in the blood sample and in the patient. Bacterial contamination is still presumed in the case of negative cultures in a septic patient with confirmed blood product contamination [67]. However, microorganisms from a positive component culture cannot be interpreted in isolation. Patients with sepsis symptoms at any time should always be investigated with blood cultures [65]. Furthermore, it is important to highlight the vulnerability of residual component cultures to secondary contamination, and results must be carefully evaluated in the clinical context [65]. The most common contaminants found in platelet units are Staphylococcus aureus or Gram-negative organisms caused by skin microbiota after needle insertion, but contaminants may also arise from an asymptomatic donor [68,69,70]. In the case of red blood cells, Gram-negative organisms (*Pseudomonas* spp., *Yersinia* spp. and *Serratia* spp.) in particular are found [71]. It should be noted that mortality increases in cases of contamination with Gram-negative organisms [71]. The treatment of the septic transfusion reaction is superposable on the treatment of sepsis and should cover the most common organisms detected associated with the septic transfusion reaction, with, in particular, a broad-spectrum antibiotic therapy [12]. There are no consensus guidelines, but antimicrobial treatment should be individualized to the local resistance patterns. A parenteral combination of vancomycin and a broad-spectrum beta-lactam or aminoglycoside may cover most likely pathogens.

### 3.11. Adverse Transfusion Reactions and COVID-19

In 2020, in Switzerland, 19 transfusion reactions were reported in patients with confirmed COVID-19 infection, of which 7 were classified as at least severe [1]. In the UK, COVID-19 was implicated in 5 TACO cases and seems to have contributed as a co-morbidity to the increase in transfusion-related deaths [6].

The rapid rise in COVID-19 infection not only had a profound impact on blood donations and blood supplies but also presented new challenges concerning blood safety and transfusion reactions [72,73,74,75,76].

Regarding transfusion safety, the transmission of respiratory viruses such as SARS-CoV by transfusion has not been reported, and initial studies already show that patients with flu-like symptoms and fever, as well as asymptomatic patients with a positive COVID-19 test result in all throat swabs, did not present viremia. Furthermore, symptomatic patients were excluded from blood donation, and therefore the risk of transfusion transmission of SARS-CoV-2 seems to be negligible [77,78]. Nevertheless, special attention must be paid to the role of platelet transfusions in COVID-19 transmission, as platelets are thought to play a major role in its pathogenesis. Hypercoagulation and thrombosis play an important part in lethality in COVID-19 patients, and recent studies show that platelets are hyperactivated in severe and non-severe COVID-19 disease [79,80]. However, platelets are not only involved in thrombosis but have also been shown to interact with pathogens such as viruses, and SARS-CoV-2 seems to bind directly to platelets [80]. Of special interest in this context is the association of SARS-CoV-2 RNA with patient platelets. Recent studies have detected SARS-CoV-2 RNA in platelets in about 25% of the examined COVID-19 patients [79,80,81]. Koupenova et al. further showed that fragmented viral genome of SARS-CoV-2 was present in platelets (but not in plasma) in all their tested COVID-19 patients. The fact that SARS-CoV-2 was fragmented, however, suggests digestion and that the protective platelet milieu may not permit viral replication. This further suggests that convalescent plasma transfusions should not contain infectious virus [82]. In this context, special attention should be paid to the case report of a 22-month-old boy who received a platelet transfusion from a COVID-19-positive donor. Five days after the donation, the nasal swab of the donor was positive for SARS-CoV-2. The recipient, however, did not show any laboratory evidence of COVID-19 infection [83]. Even though SARS-CoV-2 RNA detection has been reported in blood from infected patients, no reports have so far confirmed a viral transmission [83,84]. The transmission of pathogenic COVID-19 via platelets is, however, not excluded. Caution should be exercised, and further data are necessary.

Another challenge during the COVID-19 pandemic was and is the risk of a shortage in blood products due to rising COVID-19 infections in potential donors and large parts of the staff of donation facilities. Many countries implemented transfusion-sparing strategies, hospital reorganizations and national media campaigns. In the US, for example, during the first week of the COVID-19 pandemic, the drop in blood donations was compensated for by blood centers in non-affected areas. In Italy, a national media campaign helped to increase the number of collected whole blood units, and in Iran the implementation of the crisis system for COVID-19 (online system for coordination among blood centers, ensuring personal protective equipment, decreasing waiting time and ensuring hygienic waiting areas) helped to increase the mean number of donations [85].

Since the beginning of the COVID-19 pandemic, new treatment strategies have been evolving. The administration of convalescent plasma obtained from patients who have recovered from COVID-19 seems to be a management option with relatively few adverse events, as described in the current literature [86,87]. A retrospective analysis by Nguyen et al. showed that adverse transfusion reactions due to convalescent plasma were relatively rare, with 13 cases of 427 that were attributable to transfusion (3.1% incidence), with FNHTR (10.9%), TACO (9.1%), allergic (1.8%) and hypotensive (1.8%) reactions being the most common [75]. Nevertheless, one case report of type II TRALI after the transfusion of 2 units of adjunctive convalescent plasma was described, leading to the death of a previously healthy 59-year-old patient suffering from a severe course of COVID-19 [76]. Despite the severity of this case, no current evidence suggests a different approach. Further research, however, is needed.

Considering the fact that the COVID-19 situation is relatively new, there is still little information available on transfusion thresholds and adverse transfusion reactions in COVID-19 patients, and treatment strategies should be carefully evaluated in the clinical context [88,89].

### 3.12. Rare Transfusion Reactions

#### Acute Pain Transfusion Reaction

Acute pain transfusion reactions (APTR) are not only rare but also poorly understood. Symptoms may include severe chest, back or proximal extremity pain, tachypnea and/or dyspnea, hypertension and tachycardia after or during RBC transfusion. A multicenter retrospective analysis found 12 reports of APTR in 29,000 analyzed medical records, and only a few case reports exist [90]. APTR is typically self-limited; treatment involves symptomatic control with pain medication, supplemental oxygen and emotional support [91].

### 3.13. Prevention of Transfusion Reactions

The best way to prevent transfusion complications is to avoid unnecessary blood transfusions by respecting the transfusion thresholds. It is imperative to be aware of the indications for blood transfusion use such as symptomatic anemia, acute sickle cell crisis and acute blood loss of more than 30% of blood volume [92]. A restrictive transfusion threshold most commonly uses a transfusion threshold of 7.0 g/dL to 8.0 g/dL [93]. There is even good evidence of a threshold of <7.0 g/dL in most clinical situations [94]. For selected situations such as myocardial infarction, chronic cardiovascular disease, neurological injury or traumatic brain injury, stroke, thrombocytopenia and cancer or hematological malignancies, including chronic bone marrow failure, however, data are still unclear and clinical judgement is indispensable [94]. 

We suggest the following thresholds based on results from clinical trials and authors’ opinions: for pre-existing coronary artery disease: 8 g/dL; for acute coronary syndromes, including acute MI: 8 to 10 g/dL; for ICU patients who are hemodynamically stable: 7 g/dL; for gastrointestinal bleeding in hemodynamically stable patients: 7 g/dL; for orthopedic surgery 8 g/dL; for cardiac surgery 7.5 g/dL; for ambulatory oncologic patients: 7–8 g/dL; and according to symptoms in palliative settings [95,96,97,98,99,100,101,102].

Several pre-transfusion safety measures should be known and applied. Pre-, peri- and post-transfusional control of vital signs is imperative. The most important measure is clearly to respect the ABO antigens and the rhesus system. It is therefore mandatory to carry out pre-transfusion tests. For non-emergency indications, a transfusion during working hours allows a full complement of staff for follow-up and ensures safety. Patient or blood product mix-ups can have fatal consequences. When in doubt, the process must be stopped immediately [103]. Furthermore, additional safety measures are carried out by transfusion centers, including risk factor screening through a donor medical questionnaire, systematic leukoreduction and routine infection screening that varies by region.

### 3.14. Hemovigilance Reporting

Hemovigilance reporting provides epidemiological surveillance, control and prevention of adverse transfusion events. The results of these reports contribute to improving patient safety and provide learning opportunities for physicians.

In Switzerland, reports of all adverse events must be addressed to Swissmedic, the Swiss Agency for Therapeutic Products. Following the Medicinal Products Licensing Ordinance (MPLO) of 14 November 2018 (status as of 28 January 2022) “any person who professionally dispenses therapeutic products or administers them to humans or who is entitled to do so as medical personnel must notify Swissmedic of any serious or previously unknown adverse effects and incidents, clusters of events, observations of other serious or previously unknown facts and quality defects that are of significance for drug safety” [104]. This applies to transfusion reactions, transfusions of incorrect blood products, quality defects and near misses. Swissmedic may be notified as early as possible or by adhering to the following time frames: deaths and clusters of events must be reported immediately on becoming known (an initial report can also be made orally) and within a maximum of 15 days, serious transfusion reactions (grade 2–4 without alloimmunization) within a maximum of 15 days and all other reports within a maximum of 60 days. In Switzerland, each hospital that is authorized to perform blood transfusions must appoint a person who is responsible for hemovigilance (RPHv). In clinical settings, the hemovigilance report is usually submitted to the RPHv by the physician responsible for the transfusion or the medical staff involved in the transfusion [105]. Hemovigilance reporting systems may differ from region to region. The UK accepts reports of serious adverse events and reactions on their SHOT website [6]. In France, hemovigilance reports are submitted online via the website of the Réseau National d’Hémovigilance Déclaration et Gestion des évènements indésirables transfusionnels, known as e-FIT [106]. Hemovigilance reports in Germany are addressed to the Paul Ehrlich Institute, the Federal Institute for Vaccines and Biomedicines, via email or mail, and the necessary document can be found on their website [107].

### 3.15. Blood Transfusion Quality

A major concern in blood safety is the storage of blood products. In Switzerland, RBCs are stored for up to 49 days, platelets for up to 7 days and FFP for up to 2 years.

Storage lesions refer to morphological, functional and metabolic changes in RBCs due to their storage. The impact of the age of blood products on quality is still a matter of debate. Although several studies failed to show benefits from the transfusion of fresher blood, concerns regarding storage lesions persist. In vitro studies have shown physiological changes due to RBC storage, such as lactic acid accumulation, a decrease in pH, ATP and 2,3-diphosphoglycerate, an increase in cell membrane rigidity, the release of inflammatory mediators and impaired nitric oxide metabolism [108,109]. Platelets, however, are stored at room temperature (20 to 24 °C), which limits the shelf life to 7 days and makes them prone to bacterial contamination. Prevention involves testing for bacterial contamination and photochemical pathogen inactivation [109].

FFP is frozen at −18 °C within 8 h of collection, which extends the shelf life to 1 year, but it can be further extended to 7 years when the FFP is stored at −65 °C [110].

In order to prevent adverse transfusion reactions, blood products are further processed.

A major role in transfusion safety is played by universal leukocyte reduction (ULR), which removes the WBCs of the donor in order to prevent WBC apoptosis and necroses and cytokine release. ULR is widely implemented in Europe and was introduced in France in 1998, in Switzerland in 1999, in the UK in 1999 and in Germany in 2001. ULR has been proven to reduce the frequency and severity of NHFTR, the risk of cytomegalovirus transmission, the risk of HLA isoimmunization and organ dysfunction in patients sustaining cardiovascular surgery [111].

Further processing measures include washing (removal of the majority of plasma proteins, electrolytes and antibodies for patients with severe allergic reactions or hyperkalemia and patients with documented IgA deficiency if an IgA-deficient donor is unavailable), irradiation (prevention of viable T-lymphocytes proliferation for patients at risk of transfusion-associated graft-versus-host disease), volume reduction (in patients with cardiac dysfunction or at risk of TACO), plasma storage in platelet additive solution (PAS) (to reduce allergic reactions) and solvent–detergent treatment of plasma derivates (which inactivates lipid-enveloped pathogens and is indicated for patients with acquired deficiency in liver disease or patients undergoing liver transplantation or cardiac surgery, and for plasma exchange in patients with thrombotic thrombocytopenic purpura) [109].

In Europe, blood transfusion safety is ensured by the “20th Edition of the Guide to the preparation, use and quality assurance of blood components” prepared by the European Directorate for the Quality of Medicines & HealthCare (EDQM) and the Commission of the European Union (EU) [112]. The guidelines are regularly updated by the European Committee on Blood Transfusion and can be downloaded from their website at https://www.edqm.eu/en/blood-guide (accessed on 8 May 2022) [113]. The guidelines give detailed instructions on donor selection, collection of blood and blood components, the processing, storage and distribution of blood components and further testing such as screening for transfusion-transmissible infections, and information is provided on the administration of blood components, as well as on associated documentation and much more [112].

## 4. Conclusions

Any symptom occurring within 24 h of a blood transfusion should be considered a transfusion reaction until proven otherwise, with most adverse events occurring within 15 min–6 h of transfusion. There is significant overlap between the manifestations of mild transfusion reactions and the early stages of a severe transfusion reaction. Therefore, until proven otherwise, a severe reaction should be suspected, and the initial management of all such transfusion reactions should follow the same steps. The first step is to stop the transfusion and maintain venous access with isotonic saline. Depending on the severity and progress of the diagnosis, specific cardiac, respiratory and renal support should be initiated. Blood product labeling and patient identification should be checked, and every incident must be reported to the transfusion center. The cornerstone of transfusion reaction prevention is a restrictive attitude towards transfusion indications and pre-transfusion testing. Transfusion reactions in COVID-19-positive patients need further research. Despite the rising trend of hemovigilance reports, especially in Switzerland in 2020, there is still an important variation in reporting rates and a wide disparity in the frequency and quality of reporting across Switzerland. This corresponds to the trend in Germany, where not all reports of adverse reactions and events have been considered, due to insufficient data quality. This trend was superposable on previous years and highlights the importance of detailed documentation, especially when death occurs due to a suspected transfusion reaction. Sensibilization of medical staff to the importance of hemovigilance must be continued.

## Data Availability

Not applicable.

## Supplementary Materials

The following supporting information can be downloaded at: https://www.mdpi.com/article/10.3390/jcm11102859/s1. Supplementary S1 Table S1: Imputability in Switzerland; Supplementary S1 Table S2: Imputability in France; Supplementary S1 Table S3: Imputability in the United Kingdom; Supplementary S1 Table S4: Imputability in Germany; Supplementary S1 Table S5: Severity of transfusion reactions. Supplementary S2 Table S1: Transfusion reactions Switzerland per imputability category in 2020; Supplementary S2 Table S2: Transfusion reactions Switzerland per severity category in 2020; Supplementary S2 Table S3: Transfusion reactions in France in 2020; Supplementary S2 Table S4: Transfusion reactions Germany in 2020.
