# Supplementary material for: Blood Transfusion Reactions—A Comprehensive Review of the Literature including a Swiss Perspective
Source: J Clin Med. 2022 May 19;11(10):2859. doi: 10.3390/jcm11102859 (PMC9144124; doi:10.3390/jcm11102859)
Supplement: Supplementary file 1 [file jcm-11-02859-s001.zip › jcm-1680747_Supplementary S1.pdf]

Supplementary S1

Table S1: *Imputability in Switzerland. Available online: https://www.swissmedic.ch/swissmedic/fr/home/humanarzneimittel/marktueberwachung/haemovigilance/haemovigilance-publications-events/haemovigilance-report-2020.html (accessed on 31 january 2022).*

**Imputability in Switzerland**		
0	Not assessable	There is insufficient or contradictory information and it is impossible to obtain supplementary information or check
1	Unlikely	The reaction is definitely/more likely to be due to other causes.
2	Possible	The reaction can be explained both by the transfusion and by other causes.
3	Probably	The reaction does not appear to be due to another cause.
4	Definite	In all probability the reaction was caused by the transfusion.

Table S2: *Imputability in France. Available online https://ansm.sante.fr/uploads/2021/12/08/20211208-rapport-hemovigilance-2020-vf.pdf (accessed on 23 march 2022).*

**Imputability in France**		
N/E	Not assessable	Insufficient data
0	Excluded/unlikely	After termination of the investigation, the reaction is definitely due to other causes.
1	Possible	The data is not sufficient to attribute the reaction either to the transfusion or to other causes once the investigation is 'completed'.
2	Probably	Available evidence clearly supports the attribution of the reaction to the transfusion, once the investigation is 'complete';
3	Definite	Evidence that cannot be doubted and that clearly attributes the reaction to the transfusion once the investigation is 'completed'.

Table S3: Imputability in the United Kingdom. Available online: https://www.shotuk.org/wp-content/uploads/myimages/Interactive_SHOT-REPORT-2020_V2.1.pdf (accessed on 26 march 2022).

**Imputability in the UK**		
N/E	Not assessable	Insufficient data for imputability assessement.
0	Excluded/unlikely	Conclusive evidence beyond reasonable doubt for attributing the adverse reaction to causes other than the blood or blood components or where the evidence is clearly in favour of alternative causes
1	Possible	Evidence is indeterminate for attributing the adverse reaction either to the blood or blood component or where there may be alternative causes
2	Probably	Evidence is clearly in favour of attributing the adverse reactions to the blood or blood component
3	Definite	When there is conclusive evidence beyond reasonable doubt

Table S4: *Imputability in Germany. Available online: www.pei.de/haemovigilanzbericht (accessed on 26 march 2022).*

**Imputability in Germany**		
-	Not assessable	Data situation insufficient because, for example, data on donor or recipient are no longer available.
-	Excluded/unlikely	Data, temporal relationship, underlying disease exclude or argue against the transfused blood component as the cause of the reaction.
-	Possible	Clinical course of the reaction and temporal relationship with the transfusion suggest transfusion as the cause, but other factors, such as the patient's underlying disease, a known septicemia before the transfusion, another source of contamination, cannot be ruled out with certainty as influencing factors or as the cause of the reaction.
-	Probably	Clinical course of the reaction and data suggest transfusion as the cause of reaction, but the data are not conclusive because, for example, a comparative antibiogram of the bacterial strain found in the blood product and the recipient is lacking, evidence of sequence homology of the virus found in donor and recipient, or evidence of corresponding antigens or antibodies could not be provided for lack of test material.
-	Definite	Clinical course of the reaction and laboratory data support the association.

__Table S5:__ Severity of transfusion reactions via Swissmedic. Available online: https://www.swissmedic.ch/swissmedic/fr/home/humanarzneimittel/marktueberwachung/haemovigilance/haemovigilance-publications-events/haemovigilance-report-2020.html (accessed on 31 january 2022).

**Severity of transfusion reactions**	
1	Non-severe
2	Permanent damage or permanent risk. If the following symptoms or findings are present, a transfusion reaction should be classified at least as severe:<br>– Allo-immunizations<br>– Fever >39°C and >2°C increase<br>– Dyspnoea / hypoxia (other than a very mild form), pulmonary oedema<br>– Loss of consciousness, drop in blood pressure (other than a very mild form)<br>– Suspected hemolytic transfusion reaction<br>– Suspected bacterial contamination / infection as a result of the transfusion<br>– Positive blood cultures in patient or blood product<br>– Timely intervention is necessary to avoid permanent damage or a life-threatening course
3	Life-threatening
4	Death
