# Supplementary material for: Blood Transfusion Reactions—A Comprehensive Review of the Literature including a Swiss Perspective
Source: J Clin Med. 2022 May 19;11(10):2859. doi: 10.3390/jcm11102859 (PMC9144124; doi:10.3390/jcm11102859)
Supplement: Supplementary file 1 [file jcm-11-02859-s001.zip › jcm-1680747_Supplementary S2.pdf]

Supplementary S2

Table S1 : *Transfusion reactions Switzerland per imputability in 2020 : Available online: https://www.swissmedic.ch/swissmedic/fr/home/humanarzneimittel/marktueberwachung/haemovigilance/haemovigilance-publications-events/haemovigilance-report-2020.html (accessed on 31 january 2022)*

Imputability 1: unlikely, 2: possible, 3: probable, 4: certain.

Diagnosis	Imputability 1	Imputability 2	Imputability 3	Imputability 4	Total
**Allo-immunizations**	0	88	473	635	1196
**FNHTR**	64	275	59	14	412
**Allergic TR**	0	43	99	30	172
**TACO**	0	21	52	15	88
**Infection**	39	3	0	0	42
**Hypotensive**	4	15	13	2	34
**Hemolytic transfusion reaction**	3	3	7	14	27
**Transfusion-associated dyspnoea**	4	11	3	0	18
**Platelet refractoriness**	0	1	4	5	10
**TRALI**	0	2	1	0	3
**Hemosiderosis**	0	0	0	1	1
**Total**	122	482	711	717	2032

Table S2 : *Transfusion reactions Switzerland per severity in 2020 available online: https://www.swissmedic.ch/swissmedic/fr/home/humanarzneimittel/marktueberwachung/haemovigilance/haemovigilance-publications-events/haemovigilance-report-2020.html (accessed on 31 january 2022).*

Severity 1: non-severe, 2: severe/permanent damage, 3: life-threatening, 4: death.

Diagnosis	Severity 1	Severity 2	Severity 3	Severity 4	Total
**Allo-immunizations**	0	1196	0	0	1196
**FNHTR**	281	67	0	0	348
**Allergic TR**	117	44	10	1	172
**TACO**	4	57	26	1	88
**Hypotensive**	2	27	1	0	30
**Hemolytic transfusion reaction**	2	18	4	0	24
**Transfusion-associated dyspnoea**	1	12	1	0	14
**Platelet refractoriness**	4	6	0	0	10
**Infection**	0	3	0	0	3
**TRALI**	0	0	3	0	3
**Hemosiderosis**	1	0	0	0	1
**Total**	424	1437	46	3	1910

Table S3: *Transfusion reactions in France in 2020: Available online : https://ansm.sante.fr/uploads/2021/12/08/20211208-rapport-hemovigilance-2020-vf.pdf (accessed on 23 march 2022).*

Diagnosis	Severity	Imputability 1	Imputability 2	Imputability 3	Total
**Allo-immunizations**	1	15	93	204	312
	2	1	2	2	5
	total	16	95	206	317
**FNHTR**	1	28	23	0	51
	2	3	0	0	3
	total	31	23	0	54
**Hémosidérose**	1	1	12	22	35
	2	0	0	4	4
	Total	1	12	26	39
**Allergy**	1	5	9	6	20
	2	1	0	0	1
	3	1	1	1	3
	Total	7	10	7	24
**Pulmonary edema due to overload**	1	0	4	0	4
	2	1	2	1	4
	3	1	1	0	2
	Total	2	7	1	10
**Immunological incompatibility**	1	1	3	1	5
	2	0	0	1	1
	Total	1	3	2	6
**Viral infection**	1	3	0	0	3
	2	0	0	1	1
	Total	3	0	1	4
**Hypertensive reaction**	1	3	0	0	3
	total	3	0	0	3
**Dyspnea without pulmonary edema**	1	1	0	0	1
	total	1	0	0	1
**Sickle cell hemolysis**	1	0	1	0	1
	total	0	1	0	1
**Lesional pulmonary edema**	2	0	1	0	1
	total	0	1	0	1
**Total**		65	152	243	460

Table S4 : *Transfusion reactions Germany in 2020: Available online: www.pei.de/haemovigilanzberich t (accessed on 26 march 2022).www.pei.de/haemovigilanzberich t (accessed on 26 march 2022).*

Diagnosis	Reported	Confirmed	Deaths
**Acute allergic/anaphylactic transfusion reaction Grad I/II**	114	105	0
**Acute allergic/anaphylactic transfusion reaction Grad III/VI**	213	190	0
**TACO**	107	88	1
**TRALI**	61	3	0
**Transfusion-associated dyspnoea**	49	39	0
**Hemolytic transfusion reaction**	86	21	3
**FNHTR**	165	144	0
**Others**	45	4	0
**Transfusion related bacterial infection**	31	2	1
**HCV, HIV, HBV**	13	0	0
**HEV**	8	1	0
**other transfusion related viral infection**	1	0	0
**Fehltransfusionen**	28	27	2
**Post-transfusion purpura**	2	0	0
**Total**	**923**	**621**	**7**
